# A cancer-associated, genome protective programme engaging PKCε

**DOI:** 10.1016/j.jbior.2020.100759

**Published:** 2020-12

**Authors:** Peter J. Parker, Nicola Lockwood, Khalil Davis, Joanna R. Kelly, Tanya N. Soliman, Ainara Lopez Pardo, Jacqueline J.T. Marshall, Joanna M. Redmond, Marco Vitale, Silvia Martini

**Affiliations:** a Protein Phosphorylation Laboratory, Francis Crick Institute, London, NW1 1AT, UK; b School of Cancer and Pharmaceutical Sciences, Guy's Campus, London, SE1 1UL, UK; c Cancer Research UK, Manchester Institute, Alderley Park, SK10 4TG, UK; d Barts Cancer Institute, Charterhouse Square, London, EC1M 6BE, UK; e GSK, Stevenage, Hertfordshire, SG1 2NY, UK; f Department of Medicine and Surgery, University of Parma, Parma, Italy

**Keywords:** PKCe, Cell cycle, Non-disjunction, Aurora B

## Abstract

Associated with their roles as targets for tumour promoters, there has been a long-standing interest in how members of the protein kinase C (PKC) family act to modulate cell growth and division. This has generated a great deal of observational data, but has for the most part not afforded clear mechanistic insights into the control mechanisms at play. Here, we review the roles of PKCε in protecting transformed cells from non-disjunction. In this particular cell cycle context, there is a growing understanding of the pathways involved, affording biomarker and interventional insights and opportunities.

## 1 Introduction

Taking a highly reductionist perspective on cellular controls, the mammalian cell cycle can be broken down into three types of regulatory input. The first being the ‘decision’ to divide, reflecting a dominance of growth promoting signals over inhibitory signals sustained through to the G1 restriction point, i.e. the point of no return with respect to cell cycle commitment (reviewed (Fisher, 2016)). In multicellular organisms, this entry into cycle is determined to a significant extent by the external environment (cell-cell/matrix contact, hormones/growth factors, nutrients, etc), leading to the accumulation of active CyclinD/Cdk4/6 activity, the phosphorylation of pocket proteins and the activation of E2F (reviewed (Cobrinik, 2005)), which in turn elicits the accumulation of active CyclinE/Cdk2. The second class of regulatory input relates to the timing and process of division (one cycle at a time, complete and proof-read replication, sister chromatid disjunction, etc); these controls are cell autonomous, imposed on the underlying machinery delivering division (DNA polymerases, microtubule motor driven chromosome separation, etc) and largely concern the integrity of the division itself. These autonomous properties are exquisitely exemplified by the process of origin licensing which restricts origin firing to once and only once per cell cycle (reviewed (Marks et al., 2017)). Thirdly, there are the controls imposing protective responses to pathological, internal/external challenges (DNA damage, replication stress, etc; see recent reviews(Blackford and Stucki, 2020; Ovejero et al., 2020)) impacting the progression of the division process and eliciting decisions to either delay progression, resolve problems and complete division, or abort and take an organism protective cell cycle exit strategy e.g. senescence, or apoptosis (Krenning et al., 2019; Petr et al., 2020).

The majority of literature relating to PKC isoforms and cell cycle controls concerns entry into cycle, i.e. the G0-G1 transition through to the restriction point, and there are clearly model dependent pro- and anti-proliferative behaviours for individual isoforms creating a complicated picture (reviewed (Black and Black, 2012; Poli et al., 2014)). Notwithstanding work in these *ex vivo* models, a lack of an absolute requirement for PKC family genes for cell division *in vivo* in the mouse (all knockouts are viable excepting PKCι and PKN2), suggests that for the controls operating to progress a ‘normal’ cell cycle, these proteins are not required. Furthermore, in the case of PKCι, conditional knockout in the adult does not prevent the transformation of AT2 cells in the lung by mutant Ras (Yin et al., 2019), consistent with the lack of any fundamental requirement in the cell cycle. A similar argument can be made for PKN2 where the knockout appears to display selective defects in mesenchymal tissue (Quetier et al., 2016).

With respect to the third class of regulatory inputs to the cell cycle, there are examples of PKC isoforms acting in response to stress and contributing to cell cycle arrest, e.g. the action of PKCδ contributing the DNA damage checkpoint (LaGory et al., 2010) and damage-associated apoptosis (Basu, 2003). There is limited mechanistic insight into how these PKC isoform requirements act or the nature of the proximal targets engaged. However, in the case of PKCε recent evidence has created the foundations of a deeper understanding of its engagement in a particular cell cycle setting and discussion of these processes is the focus of this review. The properties of PKCε and functions previously ascribed to this isoform in the context of cell cycle/proliferation are discussed briefly by way of background, before detailing the operation of the protective, PKCε-dependent cell cycle programme.

### 1.1 PKCε properties

**Fig. 1:**
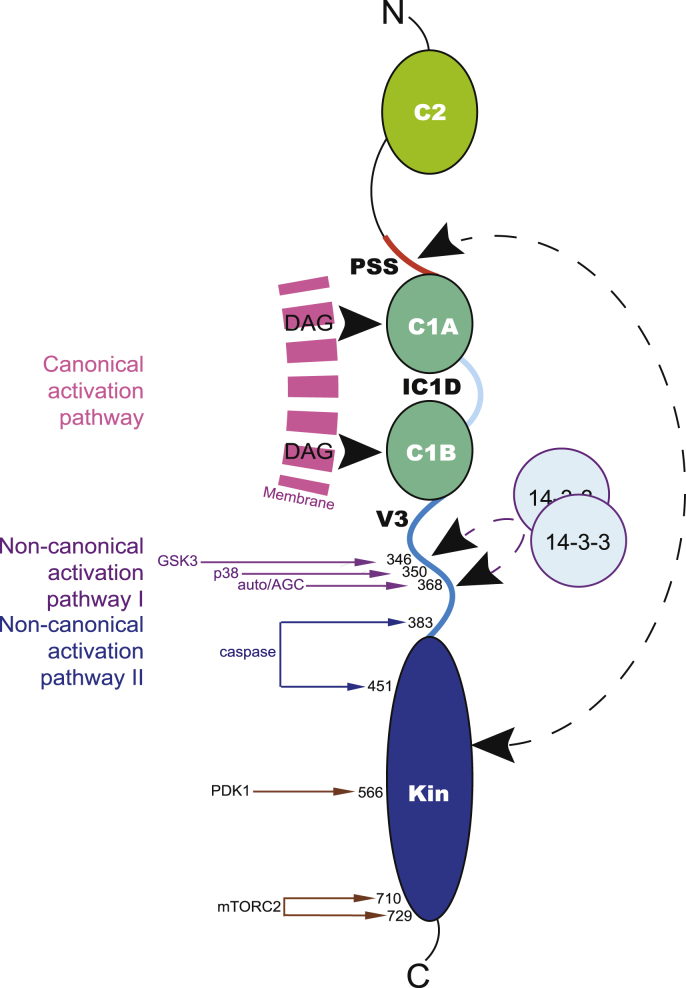
**Organisation of PKC**ε. A schematic diagram is shown of the domains of PKCε from the N-terminus (top) through the C2 domain, pseudosubstrate site (PSS), C1A domain, inter C1 domain region (IC1D), C1B domain, variable 3 region (V3) and C-terminal Kinase domain (Kin). Interacting ligands germane to the review are indicated; specifically, diacylglycerol (DAG) in a membrane compartment, 14-3-3, autoinhibition with the PSS binding the kinase domain substrate site (----). Post-translational events discussed in the text are illustrated alongside their effectors (kinases/target sites and protease/cleavage sites). Canonical and non-canonical activation pathways are indicated and colour coded. (For interpretation of the references to colour in this figure legend, the reader is referred to the Web version of this article.)

The PKCε isoform retains many of the basic properties shared with other members of this serine/threonine protein kinase family (see reviews (Mellor and Parker, 1998; Zeng et al., 2012)). The protein is organised in a modular fashion with an N-terminal regulatory domain comprising a Ca2+-insensitive C2 domain, an inhibitory pseudosubstrate site, two C1 domains (C1A and C1B) with a short intervening sequence (inter C1 domain, IC1D), followed by a V3 region that links to the C-terminal kinase domain (Fig. 1). In most cells and tissues in the basal state, PKCε is in a closed autoinhibited conformation, with the inhibitory pseudosubstrate site occupying the kinase domain substrate binding pocket. The conformation of the kinase domain that engages the autoinhibitory site is typically phosphorylated on three priming sites in the activation loop (PDK1 dependent (Dutil et al., 1998; Le Good et al., 1998);) and at the turn motif and at the hydrophobic motif at the C-terminus (both mTORC2-dependent (Cameron et al., 2011)) This closed and phosphorylated conformer, is thus inactive through steric autoinhibition, but nevertheless is in a latent active state such that on ligand/partner binding, which triggers an open conformation, activity of the kinase is expressed without the demand of upstream kinase action. This contrasts starkly with the related AGC protein kinases of the Akt family, which require acute phosphorylation by PDK1/mTORC2 for activity (see (Cameron et al., 2007)).

Canonical, ligand dependent activation of PKCε involves the ‘breathing’ of the regulatory domain-catalytic domain inhibitory interaction, such that the C1 domains can ‘sample’ membrane compartment(s) and bind their endogenous ligand diacylglycerol (DAG). The extent to which this opening of the conformation is influenced by for example C2 domain binding to proteins/membranes is not established. For PKCβ a role for the C2 domain driven by Ca2+ has been established (Nalefski and Newton, 2001). This canonical activation of PKCε is likely associated with various receptor triggered phospholipase C-dependent responses (Bunney and Katan, 2011) and pharmacologically triggered by C1-binding phorbol esters and related agents (Kazanietz, 2002). These pharmacological agents have a sustained effect on their targets as unlike DAG, they are not rapidly metabolised. Prolonged activation triggered in this manner has demonstrated that in their active states PKC family members are typically downregulated and this is in part associated with dephosphorylation and degradation (see (Newton, 2010)).

There are at least two ‘non-canonical’ pathways to activation that are pertinent to this review. The first relates to the original pioneering discovery of PKC by Nishizuka and colleagues, where it was shown that proteolysis was responsible for revealing activity in crude extracts (Takai et al., 1977). While these observations almost certainly related to the cPKC isoforms (α, β γ), it has been shown that purified PKCε can also be activated by proteolysis through cleavage in its V3 domain (Schaap et al., 1990), by inference reducing the engagement of the autoinhibitory regulatory domain and the kinase domain (Fig. 1). It has been shown that physiologically, caspase activation can also drive V3 domain cleavage (Basu et al., 2002) and this turns out to be central to an M-Phase engagement of PKCε (see further below (Kelly et al., 2020)). A second non-canonical activation pathway for PKCε involves the assembly of a PKCε-14-3-3 complex (Saurin et al., 2008). The 14-3-3 family of scaffolding proteins function as obligate dimers to interact with and control partner proteins in a highly regulated fashion, typically but not exclusively through recognition of site-specific phosphorylation (Obsilova et al., 2014). In the PKCε complex, interaction is with two distinct sites in the V3 domain (Kostelecky et al., 2009) that are phosphorylated through the action of p38 at Ser350 priming GSK3 to target the proximal Ser346 site and also an autophosphorylation/AGC kinase phosphorylation at Ser368 (Saurin et al., 2008) (see Fig. 1). Assembly of this complex appears to open the conformation of PKCε eliciting a lipid-independent activity (Saurin et al., 2008). It is notable that for both of these non-canonical mechanisms, the activated form of PKCε has the capacity to engage targets that are not necessarily constrained at the membrane.

### 1.2 PKCε suppressive and proliferative actions

Early studies associated with the ectopic expression of PKCε in adherent mesenchymal cells (NIH3T3, Rat6 cells), provided evidence that PKCε was ‘oncogenic’, in conferring an increased growth rate, growth to a higher density and growth in soft agar (Cacace et al., 1993; Mischak et al., 1993). This was associated in Rat6 cells with altered cyclin expression (Han et al., 1995). The effects in NIH3T3 cells were shown to be kinase domain dependent based upon the use of δ/ε chimera expression in a xenograft setting (Wang et al., 1998). In the LNCaP prostate cell model, ectopic PKCε was shown to trigger androgen-independent proliferation again impacting ERK activation and translation of 5′-cap-dependent mRNAs (Wu et al., 2002). In the LM3 breast cell model, PKCε expression also promotes anchorage independent growth and is pro-metastatic (Grossoni et al., 2009). In the GH3B6 pituitary tumour cell line, PMA-induced proliferation has been shown to be associated with PKCα and ε activation correlating with activation of ERK pathways (Petiti et al., 2010), although it is noted that all isoforms have the capacity to trigger ERK pathway activation (Schonwasser et al., 1998). Consistent with a growth promoting behaviour, miRNA-146a is a reported tumour suppressor and has been found to bind to PKCε mRNA and reduce protein expression associated with inhibition of proliferation in a papillary thyroid carcinoma model (Zhang et al., 2014). Down-regulation of PKCε protein by phorbol esters can also impact proliferation through loss of protective mechanisms as observed in primary AML cells and their sensitisation to TRAIL induced apoptosis (Gobbi et al., 2009).

In mouse models, there is evidence of cancer promotion activity associated with ectopic PKCε expression. Overexpression of PKCε in the epidermis sensitises to PMA-induced carcinoma formation effectively by-passing the papilloma stage (Reddig et al., 2000). Probasin promoter-driven expression of PKCε in the mouse prostate elicits a typical preneoplastic prostate lesion (Benavides et al., 2011). In this model, the additional deletion of PTEN synergises in tumour formation, acting through a CXCL13/CXCR5 autocrine mechanism (Garg et al., 2017).

Interestingly, inhibition of proliferation and cell cycle arrest associated with PKCε action has been reported in a number of contexts, in part linked to cell cycle inhibition associated with late G1 treatment with low doses of the tumour promoter PMA (Huang and Ives, 1987). In lymphoblastoid cells, this inhibition of proliferation in response to low dose PMA, was shown to be sensitised by ectopic expression of PKCε (Mihalik et al., 1996). A similar effect was observed in rat 3Y1 cells, although PKCα, δ and ε all sensitised to PMA in this setting acting through inhibition of E2F (Nakaigawa et al., 1996). Low density NIH3T3 cells are also sensitive to PMA-induced inhibition of cell cycle progression and here it has been reported that PKCε mediates this effect through induction of the Cdk inhibitor p21 (Petrovics et al., 2002). Contrary effects on p21 have been reported in non-small cell lung cancer lines based on the use of siRNA and dominant negative kinase dead PKCε approaches (Bae et al., 2007), although it is noted that the mutational manipulation associated with mutations in the conserved lysine can have pleiotropic effects (Garcia-Paramio et al., 1998). Interestingly the inhibition of proliferation through PKCε has been observed also in normal haematopoiesis (Bassini et al., 1999; Gobbi et al., 2013) and in restoring differentiation of hematopoietic progenitors from primary myelofibrosis patients (Masselli et al., 2015).

These studies illustrate the extremes relating to PKCε in terms of cell cycle controls, proliferation and transformation. It is likely that the variations in behaviour reflect a combination of the high level of expression of the ectopic protein, with the potential for non-specific effects on cell cycle behaviour (acknowledging that such variations can occur in pathological settings), and the cell-specific wiring of cell cycle controls in divergent models under different growth conditions. Whilst expression changes in PKCε have been described in cancer transcriptomic studies, evidence that any of these effects plays out functionally in cancer patients is missing and this is reflective of a paucity of mechanistic insight into specific PKCε actions for which biomarkers might then inform on its action in disease states.

### 1.3 A PKCε dependent genome protective programme

**Fig. 2:**
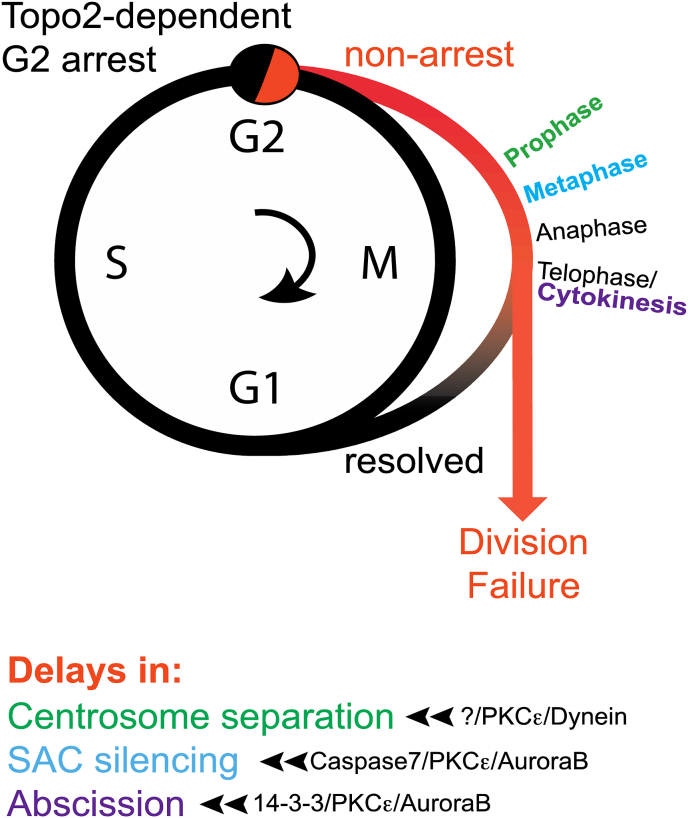
PKCε action in transformed cells. The Topo2-dependent arrest in G2 is indicated diagrammatically in the context of a normal cell cycle. The switch to a PKCε-dependent pathway (red) occurs in cells failing to arrest (see text). This leads to a series of delays under the control of PKCε as indicated. These are implemented by distinct mechanisms (see text for a more detailed discussion). Resolution of non-disjunction stress enables the maintenance of chromosomal integrity and the completion of division; failure to resolve produces division failure and/or chromosome damage. (For interpretation of the references to colour in this figure legend, the reader is referred to the Web version of this article.)

In a subset of transformed cell models, three points of dependence on PKCε have been described that serve to delay cell cycle progression and protect from sister chromatid non-disjunction (Fig. 2). The common property defining dependence on PKCε for all three actions and prompting the notion of an alternative regulatory programme, is the inability to execute a Topoisomerase2(Topo2)-dependent G2 arrest apparent on treatment with the Topo2 inhibitor ICRF193 (note this is not a Topo2 poison). This arrest has been referred to as a G2 checkpoint (Downes et al., 1994), however it is perhaps more accurately described as a Topo2A dependent G2 arrest. The implementation of this arrest is dependent upon a set of genes that are distinguished from those engaged in DNA damage responses and involves inter alia the action of the SMC5/6 complex triggering SUMOylation of Topo2A at a C-terminal site (Deiss et al., 2019). Loss of SMC5/6 or associated subunits, or in the case of a rare patient mutation, inactivation of the NSE2 subunit, prevents the normal G2 arrest on ICRF193 treatment (Deiss et al., 2019).

Associated with dysfunction of this arrest pathway in transformed cells, is a characteristic progression through cycle despite retention of non-disjoined, catenated sister chromatids, problems exacerbated by the additional loss of PKCε activity (Brownlow et al., 2014). The sensing mechanisms have yet to be defined, but the engagement and requirement for PKCε are interpreted as supporting protective actions in delaying cell cycle progression, to effect resolution from non-disjunction and hence the successful completion of cell division.

### 1.4 The aurora B abscission checkpoint

The initial evidence relating to the action of PKCε in this checkpoint arose from the finding that PKCε was phosphorylated in a cell cycle dependent manner at Ser346 (Saurin et al., 2008). This was found to be part of a series of V3 domain phosphorylations involved in the assembly of a 14-3-3 complex, a structure later resolved (Kostelecky et al., 2009) and a complex which was shown to be required for the efficient completion of cytokinesis in HeLa cells (Saurin et al., 2008). Subsequent evidence demonstrated that PKCε acted at cytokinesis to enable exit from the well characterised Aurora B abscission checkpoint (Pike et al., 2016).

The abscission checkpoint is typically engaged when there is DNA sensed in the cleavage furrow (recently reviewed (Petsalaki and Zachos, 2019); (Nähse et al., 2017)). This checkpoint is dependent on Aurora B activity for its implementation and inhibition of Aurora B prior to arrest prevents checkpoint engagement and leads to completion of daughter cell separation associated with extensive DNA damage; inhibition post-implementation of the checkpoint, leads to furrow regression and failure to divide, indicating that Aurora B activity is required also for efficient exit from the checkpoint (Steigemann et al., 2009). PKCε appears to act in this second phase of Aurora B action and does so through the direct phosphorylation of Aurora B on Ser227 in the activation loop of the kinase (Pike et al., 2016). Intriguingly this switches the substrate recognition behaviour of Aurora B such that it now has a much greater propensity to phosphorylate Borealin at Ser165; elimination of this phosphorylation on Borealin phenocopies PKCε inhibition providing strong evidence that this lies on the pathway of checkpoint exit (Pike et al., 2016).

Aurora B is the catalytic component of the chromosome passenger complex (CPC) alongside the adaptor proteins INCENP, Borealin and Survivin (reviewed (Kitagawa and Lee, 2015)). Abscission checkpoint engagement involves Aurora B phosphorylation of CHMP4C at Ser210 (Carlton et al., 2012); Borealin in turn can interact with the ESCRTIII machinery protein CHMP4C (Capalbo et al., 2016). The checkpoint-associated delay to completion of cytokinesis appears to reflect the cooperation of CHMP4C with ANCHR in the sequestration of Vps4, which is required for completion of abscission at the abscission zone (Thoresen et al., 2014). It has been suggested that the inactivation of Aurora B is involved in the release of Vps4, however based upon the requirement for PKCε acting via Aurora B and Borealin it is likely that it is this switch in Aurora B activity that is involved in the release of Vps4, although this has not been established. Recent evidence indicates that the PP1 dependent dephosphorylation of CHMP4C S210 counteracts Aurora B action at this site, enabling checkpoint exit once DNA bridging has been resolved (Bhowmick et al., 2019).

The requirement for 14-3-3 complex formation is clear in this process and this has been interpreted in respect of the lipid-independent activity associated with the assembly of this complex (as discussed above). This imples that the action of PKCε at the midbody may be independent of its membrane recruitment. This has yet to be resolved as indeed has the requirement of 14-3-3 for the midbody phosphorylation of Aurora B at Ser227; does this complex sit on the defined Aurora B phosphorylation pathway reflecting a necessary and sufficient output controlling abscission, or are there multiple parallel required outputs that have yet to be uncovered?

### 1.5 Delayed SAC silencing

The increase in anaphase irregularities associated with PKCε loss of function in ICRF193 non-arresting transformed cells (e.g. the higher frequency of PICH positive ultrafine bridges), indicated that even prior to its engagement in the abscission checkpoint, there was an exacerbation of underlying problems when PKCε is inhibited or knocked-down. This led to the observation that in such cells, PKCε controlled the timing of SAC silencing, delaying this by many tens of minutes and correlating with an increase in catenated sister chromatids in metaphase (Brownlow et al., 2014). This delay in anaphase entry is associated with the retention of BubR1 at kinetochores, affording a transient BubR1high/Mad2low kinetochore biomarker of the imposed delay. The retention of BubR1 may in part explain the delay, given its role in the control of APC/C and hence onset of anaphase (see (Kapanidou et al., 2015)).

More recently the mechanism of PKCε engagement in this process has been addressed and this is of particular interest given the chromatin-associated changes involved in the SAC silencing delay and the conventional activation of PKCε in membrane compartments (see above). Notably in the context of this compartmental issue, it has been reported that there is a constitutive cell cycle dependent activation of PKCε in a chromatin sub-compartment that is effected by V3 domain cleavage (Kelly et al., 2020). Proteolysis occurs principally through Caspase 7 acting at Asp383 (the critical, activating V3 domain site) and Asp451 (kinase domain site), in line with earlier caspase site mapping work (Basu et al., 2002). This non-apoptotic caspase action is absolutely required for the delay to anaphase entry and mutation of the PKCε Asp383 caspase cleavage site blocks any delay to anaphase onset in a manner that is artificially reversible through regulated cleavage of an engineered TEV site or via expression of the kinase domain of PKCε but not the full length non-cleavable protein (Kelly et al., 2020).

The kinase domain generated in this membrane-independent M-phase process triggers downstream events that act to promote resolution of chromatin catenation, based on the extent of retained sister chromatid catenation, and also to impose the delay to checkpoint silencing, as determined by the timing of anaphase entry (Kelly et al., 2020). Interestingly, an essential element in the actions of PKCε is again the phosphorylation of Aurora B at Ser277 (as at cytokinesis; see above) and in this context it was shown that this is critical to the activation of Topo2A through a switch in Aurora B phosphorylation of Topo2A (Kelly et al., 2020). There are however other targets that are necessary for this arrest/resolution function based upon a genetic code expansion-based substrate screen and knock-down (Davis, Martini and Parker unpublished). How these additional candidate targets play into this delay in anaphase entry and non-disjunction resolution remains to be determined.

### 1.6 Altered centrosome migration

Investigation of pre-mitotic events that might reflect the engagement of this programme of PKCε dependency led to the observation that PKCε inhibition or loss influences the progress of centrosome separation, impacting mitotic spindle assembly (Martini et al., 2018). It has yet to be determined what the activating triggers are for PKCε at this cell cycle stage, however it appears that the actions of PKCε are directed at cytoplasmic dynein. Thus in a cell cycle dependent manner, PKCε and dynein are found to interact as judged by co-immunoprecipitation and in situ proximity ligation assays (Martini et al., 2018). That these events are part of the PKCε protective programme is evident from centrosome migration being under PKCε control if and only if the G2 arrest is dysfunctional.

### 1.7 PKCε, an opportunity for intervention?

The requirement for PKCε to facilitate the efficient division of that subset of tumour cells with a defective Topo2-dependent G2 arrest as observed on treatment with ICRF193, suggests that this very druggable protein is a rational target in this subset of cancer settings. The potential impact is high given the prevalence of this loss in tumour cell lines (~60%). Furthermore, the finding that the knockout of the gene in the mouse is viable, promises a good therapeutic index and although there may be some emergent dependencies (e.g. in cardiac protection to ischemic events) this should be manageable in the context of a life-threatening cancer.

The exacerbating effects of ICRF193 treatment in the context of PKCε inhibition in these sensitive models begs the question of whether significant benefit might only be seen when Topo2A is inhibited. This should be a tractable question *in vivo* through use of the structurally related inhibitor dexrazoxane which is deployed clinically as a cardio-protective agent in anthracycline treatment (see (Cvetković and Scott, 2005)). Whether the *ex vivo* behaviour of these models plays out in an *in vivo* setting and indeed whether this converts into a clinical opportunity remains to be determined.

## Declaration of competing interest

None.

PJP drafted the manuscript and all authors added information, revised sections and edited the final version.
